# Prediction of Protein–Protein Interactions Based on Integrating Deep Learning and Feature Fusion †

**DOI:** 10.3390/ijms25115820

**Published:** 2024-05-27

**Authors:** Hoai-Nhan Tran, Phuc-Xuan-Quynh Nguyen, Fei Guo, Jianxin Wang

**Affiliations:** Hunan Provincial Key Lab on Bioinformatics, School of Computer Science and Engineering, Central South University, Changsha 410083, Chinaguofei@csu.edu.cn (F.G.)

**Keywords:** protein–protein interaction, machine learning, sequence embedding

## Abstract

Understanding protein–protein interactions (PPIs) helps to identify protein functions and develop other important applications such as drug preparation and protein–disease relationship identification. Deep-learning-based approaches are being intensely researched for PPI determination to reduce the cost and time of previous testing methods. In this work, we integrate deep learning with feature fusion, harnessing the strengths of both approaches, handcrafted features, and protein sequence embedding. The accuracies of the proposed model using five-fold cross-validation on Yeast core and Human datasets are 96.34% and 99.30%, respectively. In the task of predicting interactions in important PPI networks, our model correctly predicted all interactions in one-core, Wnt-related, and cancer-specific networks. The experimental results on cross-species datasets, including Caenorhabditis elegans, Helicobacter pylori, Homo sapiens, Mus musculus, and Escherichia coli, also show that our feature fusion method helps increase the generalization capability of the PPI prediction model.

## 1. Introduction

Protein–protein interactions (PPIs) are understood as physical contact via electrostatic forces or hydrophobic effects between proteins that occur in a living cell. Determining protein interactions could help in understanding the function of proteins through their activity in cells. In addition, the interaction pattern among proteins also suggests new drug designs. Thus, identifying accurate protein–protein interactions (PPIs) is crucial. Accurate determination of PPIs from large amounts of data using experimental biological methods is generally expensive and time-consuming [1]. To solve these issues, as a branch of computational methods, machine learning (ML)-based methods have been studied. In this study, we focused our attention on sequence-based approaches because of the advantages of rich and easy-to-search protein sequence data sources compared to protein structure data sources [2]. In addition, numerous tests have shown that amino acid sequence information alone is capable of identifying new protein–protein interactions [3,4,5].

In general, recent sequence-based methods have focused on identifying new feature extraction methods from sequence information, while others have focused on developing predictive models. For example, Guo et al. [6] proposed using the auto-covariance descriptors (ADs) to convert amino acid sequences within a protein into feature vectors, while other authors such as Yang [7], You [8,9], and Zhou [10] suggested using multi-scale continuous and discontinuous region encoders to transform protein sequences into feature vectors. Considering feature fusion techniques to build higher-quality features for PPI prediction, Chen et al. [11] proposed the LightGBM-PPI model and used a combination of multiple descriptors, including Pseudo-Amino Acid Composition (PseAAC), Autocorrelation (AC), and CT to capture the information in encoding protein sequences. Moreover, Yu et al. proposed two models, GTB-PPI [12] and GcForest-PPI [13], for determining PPIs, in which physicochemical, sequence, and evolutionary information was integrated into representative features of the protein.

The works mentioned above have shown that protein sequence descriptors can be widely applied to the PPI prediction problem. However, these feature extraction methods require great human effort in feature engineering. To address this issue, deep learning models have been designed to automatically learn protein sequence representation for PPI prediction. For example, Hashemifar et al. [14] proposed a convolutional neural network (CNN) to determine PPIs, which is called DPPI. According to Hashemifar’s method, a DL model was used to obtain high-level and essential feature representations from the evolutionary information contained inside the position-specific score matrix (PSSM). A few research works have demonstrated the effectiveness of ensemble learning methods in which multiple deep learning models—CNNs, RNNs, and MLPs (multi-layer perceptron neural networks)—are combined together. For instance, Stringer et al. [15] developed PIPENN, a deep ensemble architecture for predicting protein interfaces, which combines the outputs of six neural networks in one (including three models designed based on three different CNN architectures, one model based on a DNN (multiple fully connected layers), one model based on the RNN architecture, and the remaining model with a hybrid architecture that combines CNN and RNN architectures). Similarly, Gao et al. [5] designed a model (named EResCNN) that combines a residual convolutional neural network, MLP, LightGBM, XGBoost, RF, and Extra-Trees to mine high-level feature information directly from protein sequences, while Aybey and Gümüş introduced SENSDeep [16], which is a sequence-based ensemble learning model using stacking different deep neural networks, to predict PPI sites. SENSDeep includes two cascades: the first cascade combines four different types of deep neural networks, CNNs, RNNs, GRU sequence-to-sequence [17], and GRU sequence-to-sequence with an attention layer [18] that encodes protein sequences, and the second cascade includes the MLP model as a classifier.

Following the natural language processing (NLP) approach, Asgari and Mofrad [19] proposed ProtVec for protein sequence embedding and Yao et al. [20] proposed Res2vec for amino acid embedding. Both of these models utilized Word2vec [21] for embedding learning. Wang et al. [22] developed a PPI prediction model based on embedding learning and a convolution neural network, named Bio2vec. In Bio2vec, the protein sequences were segmented into subword sequences using the unigram language method [23], then a Skip-Gram model [21] was established to learn to represent a protein sequence as a numerical feature vector. Yang et al. [24] used Doc2vec [25] to learn the embedding of a pair of protein sequences and employed a random forest (RF) classifier to predict the protein interactions between humans and viruses. Considering masked language modeling and self-supervised learning, Brandes et al. [26] proposed a language model of proteins, named ProteinBERT, for predicting protein functions. In the task of identifying PPIs from amino acid sequences, ProteinBERT can be used in the feature extraction step by generating embeddings for the protein sequences as features.

It is widely recognized that success in predicting PPIs primarily relies on representing protein sequences and selecting suitable learning models. However, there are still certain difficulties and challenges in constructing a classifier that requires comprehensive and crucial feature information to predict PPIs. In addition, it is also necessary to predict PPIs more accurately and effectively using multi-information fusion. Inspired by these observations, we propose a novel PPI predictive model called DF-PPI (Deep Fusion-PPI). In our model, we employ a feature extraction step that uses three descriptors: F-vector [27], LD [7], and APAACplus (a new variant of APAAC [28] that we introduce here). To learn protein sequence embeddings, we use Doc2vec. We then propose a learning model that combines these features through deep learning to create stronger feature vectors for improving the prediction performance. We tested DF-PPI on various PPI datasets, including Yeast (core subset), Human, Caenorhabditis elegans, Helicobacter pylori, Homo sapiens, Mus musculus, and Escherichia coli, and found that our proposed model outperforms existing models. Moreover, our model shows promising performance on PPI network datasets, including the one-core network, the Wnt-related signal pathway network, and the cancer-specific network.

## 2. Results and Discussion

### 2.1. Datasets

We conducted experiments using 10 benchmark PPI datasets. The first dataset was the Yeast core dataset [29], which was taken from the DIP database [30]. Protein sequences with less than 50 amino acids and a sequence identity greater than or equal to 40% via CD-HIT [31] were removed from this dataset. This dataset consists of 5594 positive protein interaction pairs, and 5594 pairs with different subcellular localizations were selected as negative samples. The second was the Human dataset introduced by Huang et al. [32], which was downloaded from the Human Protein Reference Database. The Human dataset consists of 3899 positive protein pairs and 4262 negative protein pairs. We also used five PPI cross-species datasets downloaded from the DIP database, which include *Caenorhabditis elegans* (Celeg), *Escherichia coli* (Ecoli), *Homo sapiens* (Hsapi), *Helicobacter pylori* (Hpylo), and *Mus musculus* (Mmusc). These datasets consist of 4013, 6954, 1412, 1420, and 313 interacting pairs, respectively. Lastly, we used three PPI network datasets, namely the one-core network (CD9), the Wnt-related pathway crossover network (Wnt), and the cancer-specific network (Cancer). The numbers of samples in the three PPI network datasets are 16, 96 and 108, respectively.

### 2.2. Evaluation Metrics

We use widely applied measurement metrics [33] to evaluate our proposed model’s performance as well as compare it with other existing models. These metrics are accuracy (*Acc*), precision (*Pre*), sensitivity (*Sen*), negative predictive value (*NPV*), *F*1 score (*F*1) and Matthew’s correlation coefficient (*MCC*). The calculation of these metrics is defined in the following equations.

(1) $$\begin{array}{cc} Acc & =\frac{TP+TN}{P+N};\; \end{array}$$

(2) $$\begin{array}{cc} Sen & =\frac{TP}{P};\; \end{array}$$

(3) $$\begin{array}{cc} NPV & =\frac{TN}{TN+FN}; \end{array}$$

(4) $$\begin{array}{cc} Pre & =\frac{TP}{FP+TP};\; \end{array}$$

(5) $$\begin{array}{cc} F1 & =\frac{2\times TP}{2\times TP+FP+FN}; \end{array}$$

(6) $$\begin{array}{cc} MCC & =\frac{TP\times TN-FP\times FN}{\sqrt{P\times N\times (TP+FP)\times (TN+FN)}}; \end{array}$$

where *P* and *N* are the numbers of positive and negative samples, respectively; TP and TN are the number of positive samples and the number of negative samples correctly predicted by the model; and FP=N−TN; FN=P−TP. In addition, we use the area under the receiver operating characteristic curve (AUROC) and the area under the precision–recall curve (AUPRC) as metrics to evaluate the performance of methods. The higher the AUC value, the higher the model’s performance.

In PPI prediction problems, a high accuracy indicates that the model is reliable for identifying both interacting and non-interacting protein pairs. The sensitivity metric quantifies how well the PPI prediction model identifies actual interactions between pairs of input proteins. Meanwhile, a higher precision indicates that the model has less error when predicting positive samples. A high sensitivity alone may lead to incorrectly predicting interactions, so NPV overcomes this by providing insights into a model’s ability to identify true non-interactions. The F1 metric is the harmonic mean of precision and recall, balancing the trade-off between sensitivity and precision. Both F1 and MCC provide a balanced assessment of a model’s ability to predict both interacting protein pairs and non-interacting protein pairs [34]. AUROC and AUPRC are used as a single measure of overall performance across all classification thresholds. A high AUPRC and AUROC show that the model is highly capable of distinguishing between interacting and non-interacting protein pairs.

### 2.3. Effect of Amino Acid Embedding Vector Dimensions

The dimension of amino acid embedding is an important hyper-parameter of the DF-PPI model, directly affecting the PPI prediction performance. In this experiment, we employed a grid search method to identify the optimal dimension for the amino acid embedding vectors. The dimensions will be selected from the range [8, 16, 32, 64, 128]. The dimensions that yielded the highest performance through five-fold cross-validation on the Yeast core dataset were regarded as the optimal dimensions. In five-fold cross-validation, the dataset is randomly divided into five folds (subsets) of equal size. The model is trained on four folds and evaluated on the remaining fold. Finally, the average measurement metrics from the five evaluations are computed and used to comprehensively evaluate the model’s performance.

Table 1 shows the values of the hyper-parameters in our experiments and the optimal values for our model. It can be seen that our model achieved the best performance when the embedding dimension is 32.

### 2.4. Comparison between APAAC and APAACplus Descriptors

This experiment aims to determine the effectiveness of the proposed APAACplus as compared to APAAC. This experiment was also performed on the Yeast core dataset using five-fold cross-validation. In this experiment, we use two descriptors, APAAC and APAACplus, to extract protein sequence features. Other descriptors, LD and F-vector, are still used in this experiment. The dimension of the embedding vector is set to 32. The experimental results are listed in Table 2. These experimental results indicate that incorporating the sequential order of amino acid triplets obtained by APAACplus might enhance the accuracy of predicting PPIs.

### 2.5. Effect of Feature Fusion Models

The performance of a machine learning model can be affected by limitations in the features it uses. It is important to identify and address these limitations to improve the model’s performance. The first limitation of handcrafted features is redundancy or noise. Therefore, feature selection techniques, such as L1-regularized logistic regression [12] and ElassicNet [11,13], have improved the performance of protein interaction prediction. The second limitation of handcrafted features is the ability to represent proteins; for example, handcrafted features often capture only specific aspects of sequence information, such as physicochemical characteristics or sequence-order information. This may not fully represent the complexity of protein interactions. The main limitation of protein sequence embeddings is that they are influenced by the fixed sequence length setting. This setting may cut off a portion of the protein sequence, leading to the loss of information that could be significant for identifying PPIs. In contrast, handcrafted feature extraction methods do not need to fix the protein sequence, so handcrafted features are not affected by this limitation.

Therefore, the objective of this experiment is to assess the effect of each individual feature type and the features combined on the proposed model’s performance in predicting PPIs. The Yeast core and Human datasets were used, and the five-fold cross-validation method was utilized to perform this experiment. It is easily observed from Table 3 that the combination improved the prediction performance compared to using only one of the two feature extraction methods. This is because combining the features reduces limitations in individual types. Additionally, using the MLP architecture to learn the feature fusion also helps deal with redundant features.

### 2.6. Effect of the Channel Weight ω

To observe the impact of the channel weight, ω (Equation (13)), on the performance of the proposed model, we experimentally change the value of ω in the range [0.1, 0.3, 0.5, 0.7, 0.9], then evaluate the achieved model performance on the Yeast core dataset through five-fold cross-validation. The experimental results are listed in Table 4.

Table 4 shows that each type of feature has a certain influence on the overall performance of the model. The model’s accuracy and other performance metrics increase as the value of ω changes from 0.1 to 0.5, with the highest values achieved at ω of 0.5. However, as ω changes from 0.7 to 0.9, the model’s performance gradually decreases. Therefore, we choose ω=0.5 to set the channel weight for the proposed model.

### 2.7. Comparison with Other Protein Sequence Embedding Approaches

Following the natural language processing approach, to determine the advantages of our protein sequence embedding strategy, we compare our strategy with three other strategies, Bio2vec [22], Res2vec [20], and ProtVec, trained on the Uniref50 [19], Yang’s work [24], and ProteinBERT [26]. In this experiment, all models are evaluated on the Yeast core and Human datasets using five-fold cross-validation in the same experimental environment. We use the trained protein sequence embeddings as initial weights for the embedding layer of DF-PPI. Table 5 shows the details of the protein sequence embedding approaches. Table 6 lists the models’ performance evaluated on the Yeast core and Human datasets.

Table 6 shows that our protein sequence embedding method, based on Doc2vec [25], yields a better PPI prediction performance than the other methods. This demonstrated that our protein sequence embedding method based on semantic mining between amino acids helped the PPI prediction model better identify information from pairs of sequences as interacting or non-interacting.

### 2.8. Validation on the Yeast Core Dataset

**Figure 1:**
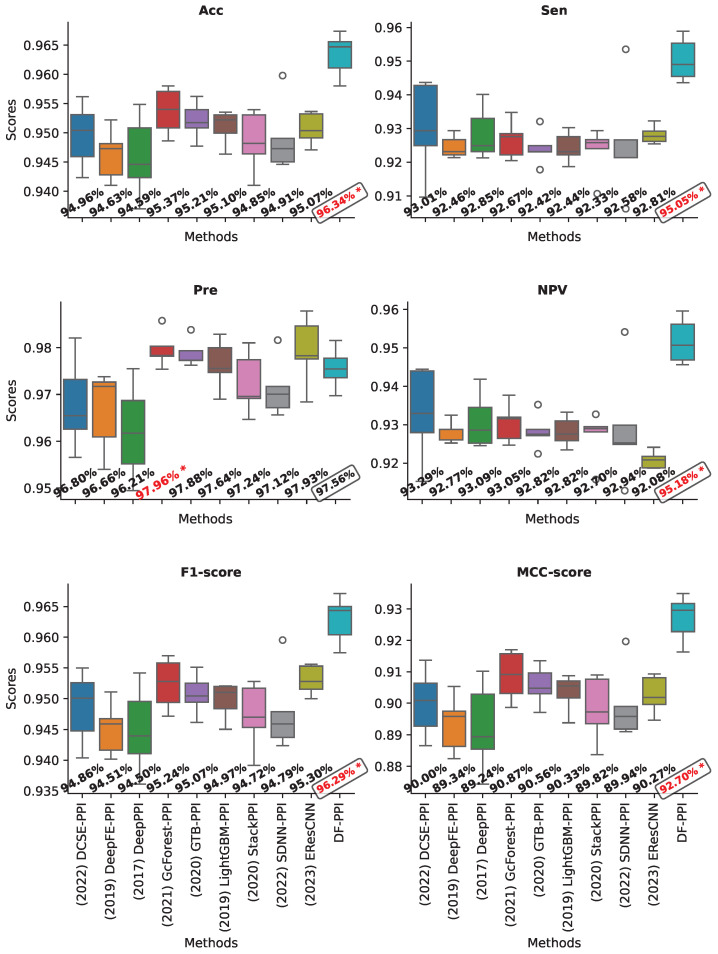
The performance of models through 5-fold cross-validation on the Yeast core dataset. The red mark “*” indicates the highest score. The box highlights the scores obtained by the proposed model.

In order to evaluate the performance of our proposed model, we compared it with the existing robust methods for PPI prediction, including DCSE-PPI [4], DeepFE-PPI [20], DeepPPI [2], GcForest-PPI [13], GTB-PPI [12], LightGBM-PPI [11], StackPPI [36], SDNN-PPI [3], and EResCNN [5]. In this experiment, we utilized the five-fold cross-validation method, which is a widely adopted method in prior research studies [4,5,13]. In addition, the optimal configuration of the mentioned models was established as described in their respective works. The performance of the models achieved on the Yeast core dataset is presented in Figure 1.

From Figure 1, it is evident that DF-PPI achieved the highest accuracy (Acc) of 96.34%, which is an improvement of at least 0.97% compared to other models. DF-PPI also has the highest sensitivity (Sen) and negative predictive value (NPV), both at 95.05% and 95.18%, respectively. This is an improvement of 2.04–3.65% and 1.89–3.55% over other models. Notably, DF-PPI had a significantly higher Sen and NPV compared to other methods. This is a crucial point because DF-PPI is less likely to incorrectly predict an interacting protein pair as non-interacting, resulting in a higher negative predictive value. Additionally, due to the model’s high sensitivity, a large number of protein–protein interactions can be accurately detected. For the specification measure (Spe), our model ranks fourth among the compared methods, at 0.4% lower than GcForest-PPI, the model with the highest specification. However, when comparing methods on harmonic measurements such as F1 and MCC, which are important measurements for a binary classifier, our model outperforms the other methods. The results show that our model outperforms the other methods with significant gains of 1.05–3.64% and 1.83–7.16% in F1 and MCC, respectively. This indicates that our model provides a harmonious balance between precision, sensitivity, and all other measures.

The stability in prediction is an important factor when evaluating model performance. As shown in Figure 1, our model demonstrates high stability across all evaluation metrics. Specifically, the DCSE-PPI model exhibits the lowest stability in terms of sensitivity (Sen) and negative predictive value (NPV), while DeepPPI is the least stable model for F1 and MCC. These findings highlight the reliability of the strategy of fusing feature types in PPI prediction.

### 2.9. Validating on the Human Dataset

**Figure 2:**
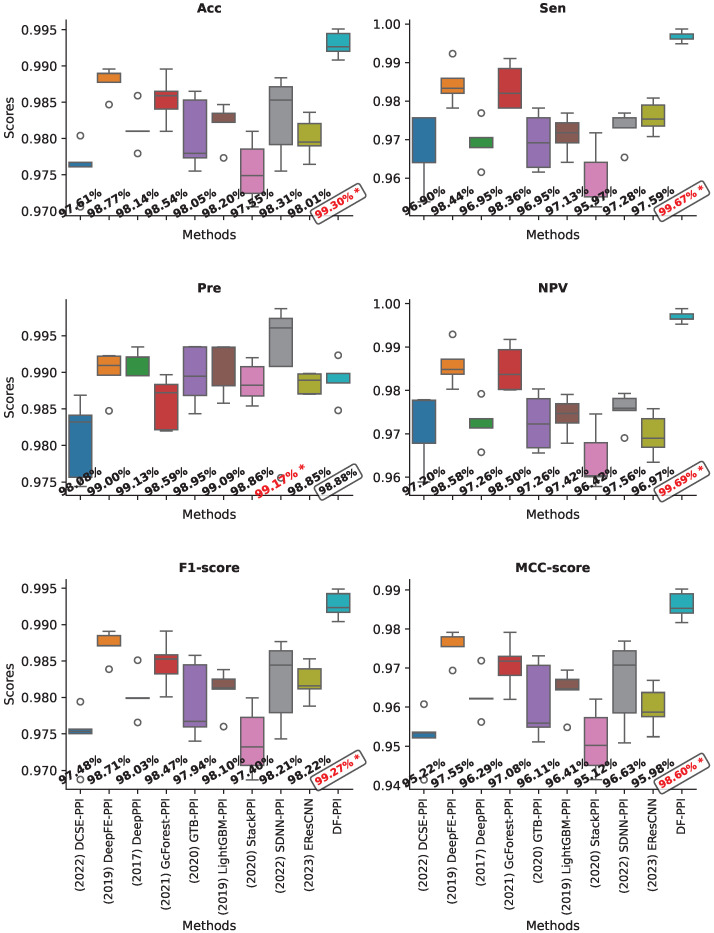
The performance of models through 5-fold cross-evaluation on the Human dataset. The red mark “*” indicates the highest score. The box highlights the scores obtained by the proposed model.

In order to evaluate the performance of the proposed model on the Human dataset, we also utilize the same experimental setup as mentioned in Section 2.8. The prediction performance of the methods is presented in Figure 2. It can be observed that our model also achieved the highest scores on most of the measures, including Acc, Sen, NPV, F1, and MCC. The obtained scores are 99.30%, 99.67%, 99.69%, 99.27%, and 98.60%, respectively. Compared with the other models, our model has an improved prediction performance by 0.53–1.69%, 1.23–2.77%, 1.11–2.49%, 0.56–1.79 %, and 1.05–3.38%, respectively.

In this experiment, our model did not achieve the highest specificity. The SDNN-PPI model scored the highest in this measure. However, SDNN-PPI is found to be unstable compared to our model in its predictions. Upon observing Figure 2, we noticed that the SDNN-PPI model has a significantly large standard deviation in the Acc, MCC, and the other measurements. On the other hand, the proposed model’s predictive stability is very high compared to the other models. In addition, the DCSE-PPI, GcForest-PPI, GTB-PPI, and StackPPI models have the highest standard deviation on the Sen and NPV metrics. This further confirms that our deep-learning-based feature-type fusion strategy is highly reliable in PPI prediction tasks.

### 2.10. Testing on PPI Cross-Species Datasets

**Figure 3:**
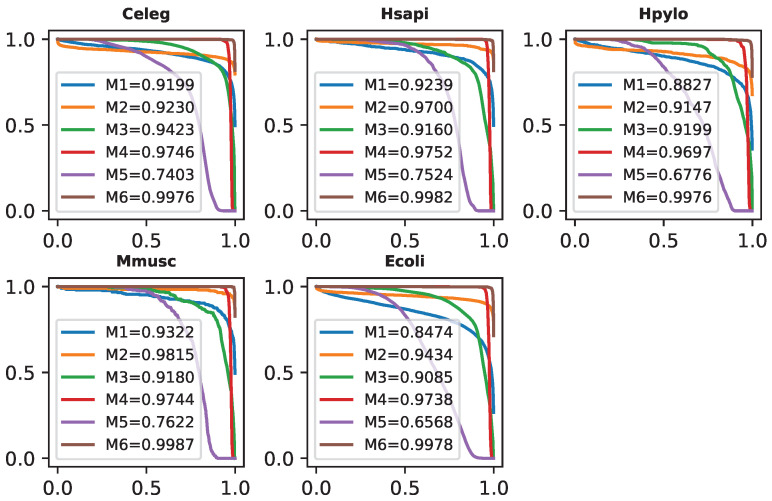
The AUC scores obtained by methods: LightGBM-PPI (M1), GTB-PPI (M2), GcForest-PPI (M3), SDNN (M4), EResCNN (M5), and DF-PPI (M6).

To validate the generalization ability of our proposed method, as well as compare it with other existing robust models, we identify PPIs of five cross-species datasets: Celeg, Ecoli, Hpylo, Hsapi, and Mmusc. For this evaluation, we used the Yeast core dataset as the training set for the models. There might be a high similarity between protein sequences in the training set and independent test sets, so we removed sequence pairs in the training set (Yeast core) that have ≥40% similarity to samples in independent test sets. After this, we obtained a new training dataset, named Yeastcore_ns (non-similarity), which includes 3877 positive and 4440 negative samples. In addition, we also removed sequences from the SwissProt database if they had ≥40% similarity to samples in the independent test sets. From there, we created a corpus, named Corpus_ns, which was used to train DF-PPI’s protein sequence embeddings. After training on the Yeast_ns dataset, the compared models are used to predict samples in cross-species datasets (containing only positive samples). As the default classification threshold of 0.5 does not help determine the certainty of the prediction of the models in this experiment, we propose to use classification thresholds in the range [0, 1] and use the area under of the accuracy curve (AUC) to compare prediction performance between models. Our method’s AUC scores in Figure 3 surpass those of other methods by a significant margin, ranging from 2.30% to 25.73%, 2.30% to 32.00%, 2.97% to 32.00%, 2.34% to 23.65%, and 2.40% to 34.10%. These results demonstrate the high generative capacity and reliability of our model for precise predictions.

### 2.11. Testing on PPI Network Datasets

**Figure 4:**
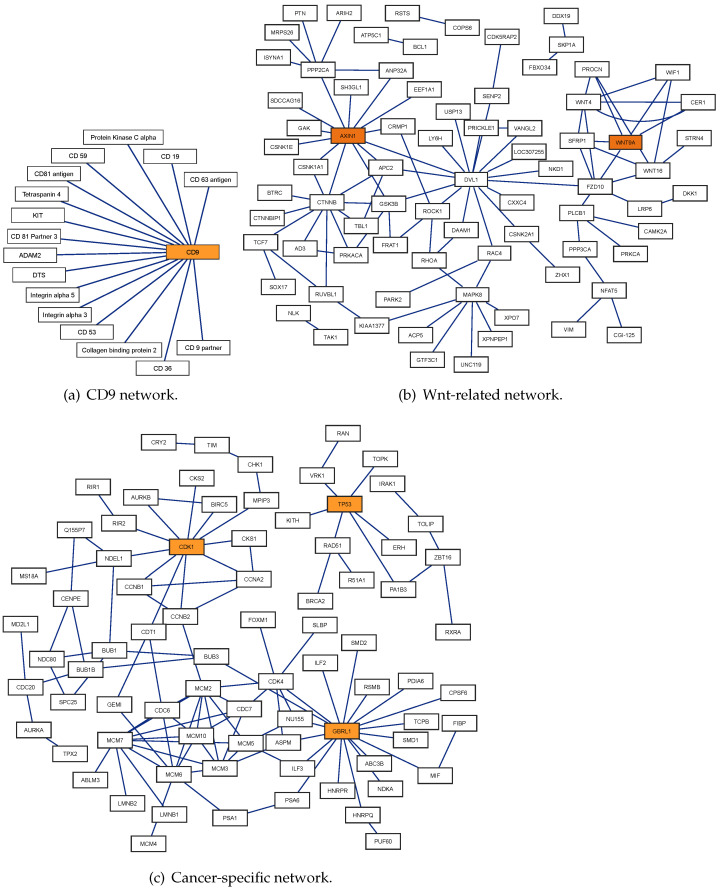
Prediction results of our model on three PPI networks.

In this experiment, we train our model on the Yeast_ns dataset, then perform prediction on three datasets PPI networks, including one-core (CD9), Wnt, and cancer networks. Testing on a PPI network is conducted to predict edges between nodes representing proteins. This task is to reconstruct the given PPI network. Reconstruction of the PPI network could significantly aid drug discovery [37,38]; for example, if a PPI is known to contribute to disease progression, a drug could be designed to break this interaction. A higher threshold ensures that the predicted interactions are more reliable for identifying drug targets. Therefore, we selected an optimal classification threshold of 0.836 for our model. To determine the threshold, the Yeast_ns dataset was split into two parts with a ratio of 8:1 for training and holdout, respectively. The optimal threshold was then selected based on the best F1 score achieved on the holdout part, within the range of [0.5, 1]. The prediction results of our model on three PPI networks are listed in Table 7 and shown in Figure 4 (the blue lines indicate the correct interaction predicted by our model).

In the experiment, as indicated in Table 7, DF-PPI accurately predicted all protein–protein interactions across all three networks. We can also see that SDNN-PPI [3] achieved correct predictions for all interactions in these networks using a classification threshold of 0.5. GcForest-PPI [13] accurately predicted all interactions in the CD9 and Cancer-specific networks; however, this model was 2.08% lower than ours in the Wnt-related network (97.92% vs. 100%). EResCNN [5] also achieved good results in reconstructing the PPI network.

CD9 belongs to the tetraspanin superfamily, and is also known as a tetraspanin protein [3]. The interaction of this protein with other proteins (Figure 4a) contributes to cell–cell interactions and tissue organization; for example, the interactions between CD9 and CD81 play crucial roles in various cellular processes and are essential for successful fertilization [39]. The success in detecting interactions between pairs of proteins in the CD9 network suggests that the DF-PPI model can help further explore the importance of CD9 in health and disease.

The Wnt-related pathway, shown in Figure 4b, is a crossover network of 77 genes with 96 interactions [36]. Among them, the WNT9A gene encodes the WNT9A protein, which plays a role in tumor formation [40]. In [41], the authors indicated that AXIN1 plays a significant role in the development of cancerous processes. WNT9A and AXIN1 are both associated with the Wnt-related pathway. A full understanding of the Wnt-related pathway may help uncover potential mechanisms and therapeutic applications [42].

As shown in Figure 4c, the cancer-specific network consists of two subnetworks. The first subnetwork comprises 64 genes with two main hubs, CDK1 and GBRL1. The pathway governed by CDK1 and GBRL1 might be critical in regulating the response of cells to stress, including DNA damage or deprivation of nutrients by controlling cell cycle progression and autophagy [43]. The second sub-network comprises 14 genes with TP53 as the main hub. TP53 gene interactions are essential for controlling the cell cycle and preventing the development of tumors by stopping cells with mutated or damaged DNA from dividing [44]. Successful prediction in a cancer-specific network is important in developing new therapeutic strategies for diseases involving cancer and the DF-PPI model could be helpful in this task.

## 3. Materials and Methods

**Figure 5:**
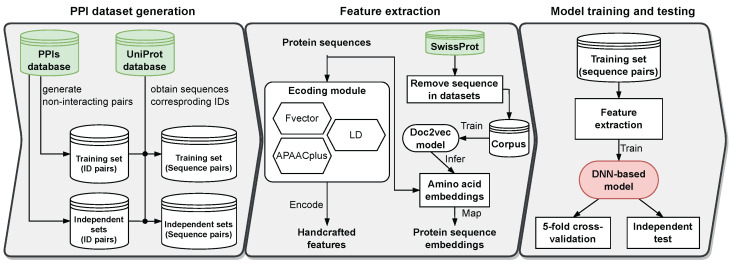
The computational pipeline for determining protein–protein interactions from only protein sequences.

We introduce a protein-sequence-based PPI prediction pipeline (Figure 5) with three stages. (1) PPI dataset generation: training/testing sets are built for model optimization and independent evaluation. Interacting pairs are from the UniProt database [45] and non-interacting pairs are from different subcellular locations. (2) Feature extraction: We extract features from protein sequences using two methods: handcrafted features and protein sequence embeddings. These features are then combined through feature fusion to improve PPI prediction. (3) Model training and testing: Our model is trained on training data and evaluated via five-fold cross-validation and independent tests. Then, extensive comparisons are made with existing robust methods.

### 3.1. Handcrafted Features

In order to convert a protein sequence into a feature vector, we employ three distinct protein sequence descriptors: F-vector descriptor, LD, and APAACplus. Notably, APAACplus is a new descriptor; we developed it based on the APAAC descriptor proposed by Chou in [28]. The use of descriptors to represent proteins as features is a well-established technique for solving the PPI prediction problem.

#### 3.1.1. Local Descriptor

**Figure A1:**
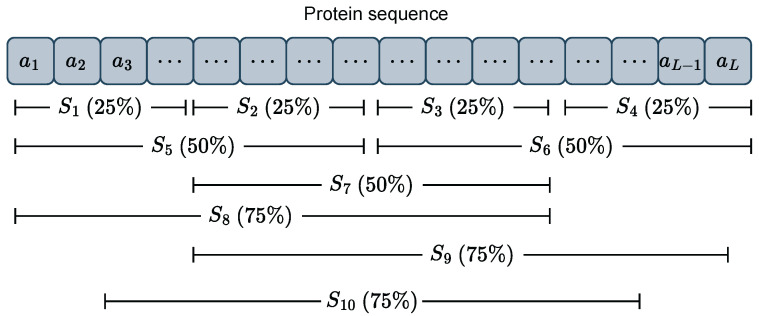
Splitting a given protein sequence into ten parts, denoted from region $S_{1}$ to $S_{10}$. $a_{i}$ indicates the $i^{th}$ amino acid after converting it to its corresponding group index.

The local descriptor (LD) was introduced by Yang et al. [7]. The LD encodes information about specific segments in a protein sequence. First, the LD divides the 20 standard amino acids into seven groups according to their physicochemical properties (Table A1). Next, the entire protein sequence is divided into ten fragments of different lengths, of which seven regions have a length that represents 25% of the given protein sequence and the remaining three regions represent 75% (Figure A1). Then, each region is extracted into features using the three descriptors of composition (C), transition (T), and distribution (D). The C descriptor represents the frequency of each amino acid group. The T descriptor reflects the conversion of an amino acid in one group to an amino acid in another group. The D descriptor describes the distribution of the amino acid groups at the beginning, quarter point (25%), midpoint (50%), three-quarter point (75%), and end of the sequence (100%). Finally, the LD concatenates the output features of C, T, and D to create a final feature vector of 70+210+350=630 dimensions.

#### 3.1.2. F-Vector Descriptor

The F-vector descriptor [27] encodes a protein sequence based on the main idea of placing amino acids on a unit circle. First, the F-vector reduces a protein sequence by classifying the 20 amino acids into separate groups. For this step, we used the amino acid classification of LD encoding Section 3.1.1. Then, the classified amino acids are further divided into four classes (denoted $G_{0}$, $G_{1}$, $G_{2}$, $G_{3}$) in the following way: four amino acid groups are selected from seven groups into one class, and the remaining three groups are selected into each remaining classes without using permutations. This classification is shown in Table A2. The process of placing amino acids on the unit circle is expressed in Equation (7). Finally, the protein sequence representation feature is calculated based on the distribution of points on the unit circle. The detailed definition for the F-vector feature is expressed by Equation (8).

(7) $$x_{j}=\cos\left(\frac{n_{j}\left(G_{k}\right)}{n\left(G_{k}\right)+1}+k\frac{\pi }{2}\right);\;y_{j}=\sin\left(\frac{n_{j}\left(G_{k}\right)}{n\left(G_{k}\right)+1}+k\frac{\pi }{2}\right);$$

where k=0,1,2,3; $n_{j}\left(G_{k}\right)$ represents the number of $G_{k}$ in the first $j^{th}$ amino acids and $n(G_{k})$ represents the number of $G_{k}$.

(8) $$F=\left.\left[f_{1}^{\left(1\right)},f_{2}^{\left(1\right)},f_{3}^{\left(1\right)},f_{4}^{\left(1\right)},\cdots ,f_{1}^{\left(35\right)},f_{2}^{\left(35\right)},f_{3}^{\left(35\right)},f_{4}^{\left(35\right)}\right.\right];$$

where $f_{1}=\frac{1}{L}\sum_{j=1}^{L}x_{j}$; $f_{2}=\sqrt{\frac{1}{L-1}\sum_{j=1}^{L}{\left(x_{j}-f_{1}\right)}^{2}}$; $f_{3}=\frac{1}{L}\sum_{j=1}^{L}y_{j}$; and

$f_{4}=\sqrt{\frac{1}{L-1}\sum_{j=1}^{L}{\left(y_{j}-f_{3}\right)}^{2}}$. *i* represents the $i^{th}$ classification among 35 ways. The F-vector generates a 140-dimensional vector to characterize each amino acid sequence.

#### 3.1.3. APAACplus Descriptor

The APAAC descriptor, introduced by Chou [28], reflects the sequence-order information and the hydrophobicity and hydrophilicity of amino acids in a protein sequence. While APAAC has been widely used in bioinformatics [2,46,47,48], it only describes the ordering relationships of amino acid pairs. The order of amino acid triads also contains valuable information not captured by APAAC. Therefore, we propose a new variant, APAACplus, that incorporates a term exploiting the sequence-order created by tripeptides. The formula for APAACplus is shown in Equation (9).

(9) $$A=\left[\frac{f_{1}}{C},\cdots ,\frac{f_{20}}{C},\frac{w_{1}\tau_{21}}{C},\cdots ,\frac{w_{1}\tau_{20+2\lambda }}{C},\frac{w_{2}\upsilon_{21+2\lambda }}{C},\cdots ,\frac{w_{2}\upsilon_{21+4\lambda }}{C}\right];$$

where $C=\sum f_{i}+w_{1}\sum \tau_{d}+w_{2}\sum \upsilon_{d}$; $f_{r}$ represents the normalized occurrence frequencies of the 20 amino acids in the input protein; $\tau_{d}$ and $\upsilon_{d}$ reflect the sequence-order correlation between all dipeptides and tripeptides in the input protein, respectively; $w_{1}$ and $w_{2}$ are two weight factors, chosen as 0.5 and 0.5 in this study; and $\tau_{d}$ and $\upsilon_{d}$ are determined via Equation (10).

(10) $$\begin{array}{c} \begin{array}{c} \tau_{d}=\left\{\begin{array}{cc} \frac{1}{L-d}\sum_{i=1}^{L-d}h_{i}^{\left(1\right)}h_{i+d}^{\left(1\right)}; & d=21,23,\cdots ,2\lambda -1\\ \frac{1}{L-d}\sum_{i=1}^{L-d}h_{i}^{\left(2\right)}h_{i+d}^{\left(2\right)}; & d=22,24,\cdots ,2\lambda \end{array}\right. \end{array}\\ \upsilon_{d}=\left\{\begin{array}{cc} \frac{1}{L-2d}\sum_{i=1}^{L-2d}h_{i}^{\left(1\right)}h_{i+d}^{\left(1\right)}h_{i+2d}^{\left(1\right)}; & d=2\lambda +1,2\lambda +3,\cdots ,4\lambda -1\\ \frac{1}{L-2d}\sum_{i=1}^{L-2d}h_{i}^{\left(2\right)}h_{i+d}^{\left(2\right)}h_{i+2d}^{\left(2\right)}; & d=2\lambda +2,2\lambda +4,\cdots ,4\lambda \end{array}\right. \end{array}$$

where *L* is the length of the protein sequence; $h_{i}^{\left(1\right)}$ and $h_{i}^{\left(2\right)}$ are the normalized hydrophobicity and hydrophilicity values for the $i^{th}$ amino acid in the protein sequence, respectively; and λ$<\frac{L}{2}$ is the maximum distance between two amino acids in a protein sequence. APAACplus generates a feature vector of 20+4λ dimensions. In this study, according to the APAAC’s default parameters, we chose λ=30, $w_{1}=0.5$ and $w_{2}=0.5$ as default values of APAACplus.

### 3.2. Protein Sequence Embedding

The hypothesis that protein sequences, akin to natural language sentences, could hold semantic information deserves further exploration as a feature extraction method. The extracted semantic information from protein sequences could be used as additional features alongside handcrafted features. Extracting semantic information could be carried out using natural language processing (NLP) techniques. In this work, we leverage Doc2vec [25], a powerful NLP technique, to extract semantic information. We constructed a training dataset of 474,326 protein sequences from SwissProt [45], excluding sequences present in our PPI datasets to avoid potential bias. We treat each protein sequence as a “document” and each amino acid as a “word”, aligning with natural language processing concepts. We adopt the PV-DM [25] (Distributed Memory Model of Paragraph Vectors) architecture, which generates both word and document vectors using stochastic gradient descent (SGD). The trained word vectors for all amino acids form a word embedding matrix, serving as pre-trained weights for the embedding layer in our PPI prediction model. Doc2vec training is implemented using the Python library Gensim [49], with default parameters.

### 3.3. The Architecture of DF-PPI

**Figure 6:**
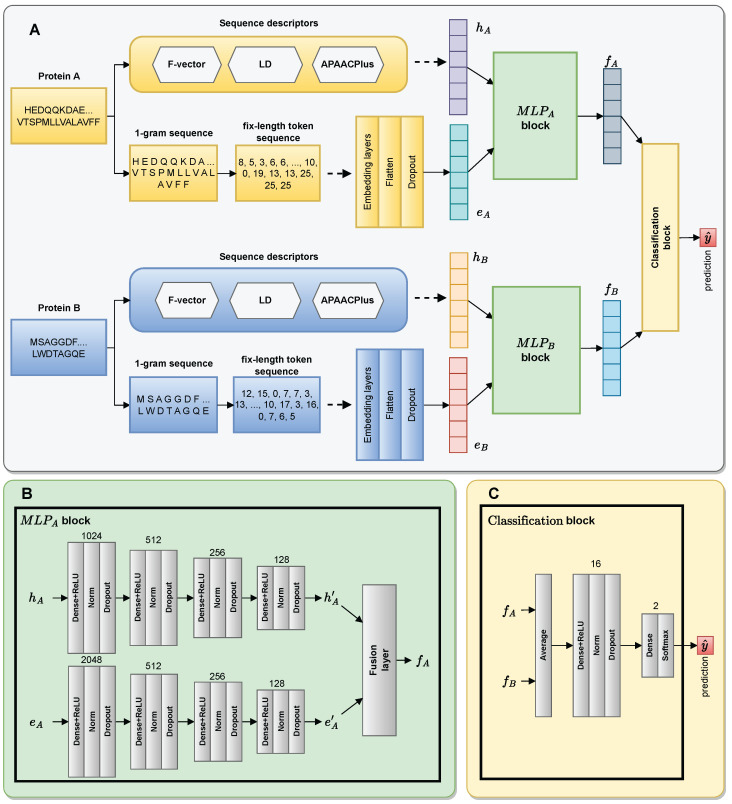
General architecture of our PPI prediction model, named DF-PPI (Deep Fusion-PPI). Part (**A**) illustrates the general architecture of the proposed model. Part (**B**) illustrates that ${\mathrm{MLP}}_{A}$’s (multilayer perceptron A) architecture and the architecture of ${\mathrm{MLP}}_{B}$ is the same. Part (**C**) illustrates the classification block.

The architecture of DF-PPI is based on a multilayer neural network (MLP). MLPs consist of multiple layers of neurons paired with non-linear activation functions, allowing them to learn complex patterns [50]. In the field of bioinformatics, many studies have successfully applied this type of neural network architecture to integrate multiple features, resulting in an improved PPI prediction performance [2,36]. In this study, the MLP architecture is employed to design the proposed model, enabling the fusion of multiple feature types for accurate predictions of PPIs. Figure 6 illustrates the architecture of our protein interaction prediction model. In the proposed model, two blocks, ${\mathrm{MLP}}_{A}$ and ${\mathrm{MLP}}_{B}$, are responsible for combining two characteristic forms of protein A and protein B, respectively, and then the interaction between these two proteins is performed by the classification block.

#### 3.3.1. Embedding Layer

**Figure A2:**
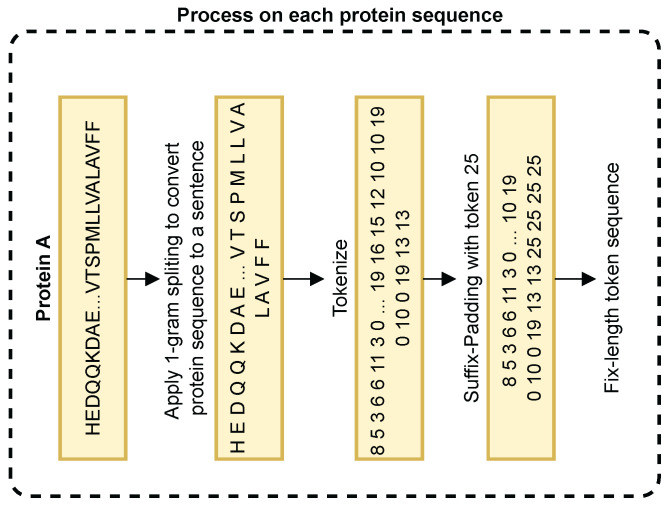
Pre-processing protein sequences into fixed-length token sequences.

By including a trainable embedding layer in DF-PPI, we have enabled it to better leverage the embedding features for identifying PPI interactions. This embedding layer requires a tokenized protein sequence as input. This is built upon our earlier work [51], where we directly convert raw amino acid sequences using a one-gram method. Due to the variation in the length of protein sequences, it was necessary to fix all sequences to the same length before they were used for the DF-PPI model. This step is accomplished using a padding technique as the following steps: (1) Set a fixed length (denoted as *N*). (2) Convert the input protein sequence to a one-gram sequence. (3) Tokenize the one-gram sequence into a token sequence. (4) Truncate the right side of the token sequence if its length is greater than N; otherwise, pad the right side of the token sequence with token 25. Tokens 0–19 represent the 20 identified amino acids, tokens 20–24 represent the 5 unidentified amino acids, and token 25 is a padding token. The padding token has a zero-vector embedding. This entire process is illustrated in Figure A2. Our previous research [51] determined the *N* value as the average length of the sequences in the training dataset. For instance, in independent tests, *N* was found to be 557.

#### 3.3.2. MLP Blocks

In DF-PPI, there are two MLPs with a similar architecture. Figure 6A illustrates the general architecture of the model. Figure 6B illustrates the ${\mathrm{MLP}}_{A}$ blocks as well as the ${\mathrm{MLP}}_{B}$ blocks in detail. The architecture of ${\mathrm{MLP}}_{A}$ is composed of two channels. The first channel consists of four dense layers with 1024, 512, 256, and 128 neurons. The second channel also includes four dense layers with 2048, 512, 256, and 128 neurons. To learn the non-linear relationship between the inputs, a rectified linear unit (relu) activation function is added after each dense layer. To speed up training and avoid overfitting, batch normalization (norm) [52] and dropout [53] layers are also added after each dense layer. Since the fourth dense layer has 128 neurons, the feature vector $f_{A}$ has a dimension of 256. Let the inputs of ${\mathrm{MLP}}_{A}$ be two vectors, $h_{A}$ and $e_{A}$, in which $h_{A}$ is pushed into the first channel and $e_{A}$ is pushed into the second channel. The outputs of ${\mathrm{MLP}}_{A}$ are two feature vectors, $h_{A}^{\prime }$ and $e_{A}^{\prime }$, and are expressed with the following formulas.

(11) $$h_{A}^{\prime }=h_{A}^{\left(4\right)};\;h_{A}^{\left(l\right)}=\mathrm{drop}(\mathrm{norm}\left(\mathrm{relu}\left(W^{\left(l\right)}\cdot h_{A}^{\left(l-1\right)}+b^{\left(l-1\right)}\right)\right);\;h_{A}^{\left(0\right)}=h_{A};$$

(12) $$e_{A}^{\prime }=e_{A}^{\left(4\right)};\;e_{A}^{\left(l\right)}=\mathrm{drop}(\mathrm{norm}\left(\mathrm{relu}\left(W^{\left(l\right)}\cdot e_{A}^{\left(l-1\right)}+b^{\left(l-1\right)}\right)\right);\;e_{A}^{\left(0\right)}=e_{A};$$

where *l* (l=4,3,2,1) denotes the $l^{th}$ hidden layer and $W^{\left(l\right)}$ and $b^{\left(l\right)}$ are the learnable weights of the $l^{th}$ layer of the neural network ${\mathrm{MLP}}_{A}$.

#### 3.3.3. Fusion Layer

Each MLP block (A and B) is composed of two channels, aiming to extract different features of the protein sequence. Then, these features are combined to represent the protein sequence via the fusion layer. On the other hand, the fusion layer also has the purpose of balancing the contribution of two types of features using the ω weight. Specifically, assuming that the weight of the handcrafted feature is ω, the weight of the sequence embedding feature is 1−ω. The two input feature vectors of the fusion layer corresponding to protein A are $h_{A}^{\prime }$ and $e_{A}^{\prime }$, so the output feature vector $f_{A}$ of the fusion layer is computed as follows:

(13) $$f_{A}=\omega h_{A}^{\prime }+\left(1-\omega \right)e_{A}^{\prime };$$

where the optimal value of ω is determined experimentally.

Similarly, for the two feature vectors $h_{B}$ and $e_{B}$ after going through the ${\mathrm{MLP}}_{B}$ block, we have the vector $f_{B}$.

#### 3.3.4. Classification Block

After the progression of two MLP blocks, we obtain two feature vectors $f_{A}$ and $f_{B}$ representing the two input proteins A and B, respectively. Each of these vectors has a size of 128. Then, the two feature vectors are fed into the classification block to calculate the interaction probability of the input protein pairs. The classification block (as shown in Figure 6C) is constructed by an average layer and two dense layers. The first dense layer consists of 16 neurons and is followed by the relu function and two other layers norm and drop. The last dense layer consists of two neurons followed by the two-class softmax function [54]. Specifically, the interaction probability is calculated as follows:

(14) $$\begin{array}{cc} f & =\mathrm{drop}(\mathrm{norm}\left(\mathrm{relu}\left(W_{1}\cdot f_{AB}+b_{1}\right)\right);\\ \hat{y} & =\mathrm{softmax}\left(W_{2}\cdot f+b_{2}\right); \end{array}$$

where $f_{AB}=\frac{f_{A}+f_{B}}{2}$; $\mathrm{softmax}\left(x\right)=\frac{\exp(x)}{\sum_{i=1}^{2}\exp\left(x_{i}\right)}$; and $W_{1}$, $b_{1}$ and $W_{2}$, $b_{2}$ are weights of the two dense layers of the classification block, respectively.

### 3.4. Training the Model

In order to train our model, we used the Adam [55] algorithm to minimize the binary cross-entropy loss function, which is defined as:

(15) $$\;L=-\frac{1}{N}\sum_{i=1}^{N}y_{i}\log\left({\hat{y}}_{i}\right)+\left(1-y_{i}\right)\log\left(1-{\hat{y}}_{i}\right);$$

where ${\hat{y}}_{i}$ is the class probability, $y_{i}$ is the actual class of the $i^{th}$ sample, and *N* is the total samples.

In addition, we utilized the time-based learning rate decay technique to reduce overfitting and increase the stability of the training process [56]. We set the parameters of the Adam algorithm to its default value, as suggested in the original article [55].

## 4. Conclusions

In this work, we proposed a model, named DF-PPI, that can leverage the power of handcrafted feature extraction and NLP-based sequence embedding techniques for sequence representation. The advantage of the handcrafted feature extraction techniques used in DF-PPI is the ability to capture protein information, such as physicochemical properties, and the amino acid distribution characteristics. Our proposed descriptor, APAACplus, has an advantage over its original, APAAC, in being able to encode information about amino acid triplets in the protein sequence. Moreover, through semantic learning, the embedding technique is capable of capturing sequence similarity, which is a type of information that can be exploited for protein representation. The goal of combining these techniques is used to enrich the available information on protein sequence feature vectors. Our results showed that this combined method improved the ability to predict PPIs compared to using only one of them. The high generalization ability of the proposed model in independent tests highlights that this combination is effective and highly reliable.

Although the proposed model achieves a high performance on most metrics, it needs improvement in terms of specificity. This might be addressed by adding negative examples that are carefully selected to represent non-interacting protein pairs to help the model see the important characteristics needed to detect negative samples. In addition, structural, gene ontology, or network-based features could be incorporated to capture the characteristics of true protein–protein interactions while minimizing noise. Combining feature types helps the model have a high generalizability; however, choosing suitable handcrafted feature extraction descriptors for PPI prediction requires a lot of effort from data scientists and biologists. In the future, we will try to reduce the complexity of the model architecture to make it easier to implement in real-world scenarios.

## Figures and Tables

**Table 1:** The performance (%) of DF-PPI using different amino acid embedding vector dimensions.

Dimension	Acc	Sen	Pre	NPV	F1	MCC	AUC	AUPR
8	95.90 ± 0.23	94.05 ± 0.39	**97.66** **± 0.29**	94.26 ± 0.35	95.82 ± 0.24	91.86 ± 0.46	98.70 ± 0.07	99.04 ± 0.04
16	96.17 ± 0.28	94.71 ± 0.49	97.55 ± 0.22	94.86 ± 0.45	96.11 ± 0.29	92.37 ± 0.54	98.86 ± 0.06	99.15 ± 0.06
32	**96.34** **± 0.34**	**95.05** **± 0.58**	97.56 ± 0.39	**95.18** **± 0.54**	**96.29** **± 0.35**	**92.70** **± 0.67**	**98.87** **± 0.08**	**99.16** **± 0.04**
64	95.91 ± 0.27	94.28 ± 0.43	97.45 ± 0.43	94.46 ± 0.38	95.84 ± 0.27	91.86 ± 0.53	98.65 ± 0.07	99.03 ± 0.05
128	96.09 ± 0.21	94.53 ± 0.25	97.57 ± 0.29	94.70 ± 0.23	96.02 ± 0.21	92.22 ± 0.42	98.73 ± 0.05	99.07 ± 0.05

Note: The bold values indicate the best values.

**Table 2:** The performance (%) of DF-PPI using APAAC or APAACplus descriptors.

	Acc	Sen	Pre	NPV	F1	MCC	AUROC	AUPRC
APAAC [28]	96.10 ± 0.20	94.76 ± 0.48	97.38 ± 0.45	94.90 ± 0.43	96.05 ± 0.21	92.24 ± 0.41	98.88 ± 0.07	99.16 ± 0.05
APAACplus (Ours)	96.34 ± 0.34	95.05 ± 0.58	97.56 ± 0.39	95.18 ± 0.54	96.29 ± 0.35	92.70 ± 0.67	98.87 ± 0.08	99.16 ± 0.04

**Table 3:** The performance (%) of DF-PPI when using the two feature types.

Metrics		Acc	Pre	Sen	NPV	F1	MCC	AUROC	AUPRC
Yeast core	H	94.33	97.25	91.26	91.78	94.15	88.85	98.04	98.51
	E	95.41	96.60	94.15	94.30	95.36	90.86	98.46	98.87
	H + E	**96.34**	**97.56**	**95.05**	**95.18**	**96.29**	**92.70**	**98.87**	**99.16**
Human	H	96.99	98.88	94.77	95.42	96.77	94.04	99.25	99.02
	E	99.11	**98.90**	99.23	99.29	99.07	98.21	99.66	99.42
	H + E	**99.30**	98.88	**99.67**	**99.69**	**99.27**	**98.60**	**99.75**	**99.57**

Note: The bold values indicate the best values. Row H—only handcrafted features, E—only protein sequence embedding, and H + E—fusion of both.

**Table 4:** The performance (%) of DF-PPI using different values of ω on the Yeast core dataset.

ω	Acc	Sen	Pre	NPV	F1	MCC
0.1	96.07 ± 0.20	94.51 ± 0.46	97.55 ± 0.23	94.68 ± 0.41	96.00 ± 0.21	92.18 ± 0.38
0.3	96.22 ± 0.24	94.82 ± 0.24	97.56 ± 0.47	94.96 ± 0.22	96.17 ± 0.24	92.48 ± 0.48
**0.5**	**96.34 ± 0.34**	**95.05 ± 0.58**	**97.56 ± 0.39**	**95.18 ± 0.54**	**96.29 ± 0.35**	**92.70 ± 0.67**
0.7	96.08 ± 0.17	94.73 ± 0.22	97.35 ± 0.23	94.87 ± 0.20	96.02 ± 0.17	92.19 ± 0.33
0.9	96.07 ± 0.32	94.83 ± 0.69	97.23 ± 0.29	94.96 ± 0.63	96.02 ± 0.33	92.17 ± 0.62

Note: The bold values indicate the best values.

**Table 5:** The details of protein sequence embedding approaches.

	Word	Sentence/Document	Embedding Vector Dimension	Training Corpus
Bio2vec [22]	Unigram ^1^	Protein sequence	32	SwissProt
Res2vec [20]	1-gram	Protein sequence	20	SwissProt
ProtVec [19]	3-gram	Protein sequence	100	UniRef50
Yang’s work [24]	5-gram	Protein sequence	32	SwissProt
ProteinBERT [26]	1-gram	Protein sequence	128	UniRef90

^1^ Generated by SentencePiece [35].

**Table 6:** The performance (%) of DF-PPI using different protein sequence embedding approaches.

Approaches	Yeast Core			Human		
	**Acc**	**F1**	**MCC**	**Acc**	**F1**	**MCC**
Bio2Vec (2019) [22]	95.47 ± 0.36	95.35 ± 0.37	91.07 ± 0.72	98.64 ± 0.14	98.57 ± 0.14	97.28 ± 0.27
Res2vec (2019) [20]	95.86 ± 0.23	95.79 ± 0.24	91.78 ± 0.46	99.25 ± 0.17	99.22 ± 0.18	98.50 ± 0.35
ProtVec (2020) [19]	96.02 ± 0.25	95.94 ± 0.27	92.11 ± 0.49	99.14 ± 0.13	99.10 ± 0.14	98.28 ± 0.27
Yang’s work (2020) [24]	95.84 ± 0.54	95.75 ± 0.57	91.77 ± 1.04	99.15 ± 0.15	99.12 ± 0.15	98.31 ± 0.29
ProteinBERT (2022) [26]	96.07 ± 0.24	95.99 ± 0.27	92.21 ± 0.45	99.08 ± 0.20	99.04 ± 0.21	98.16 ± 0.40
Doc2vec (ours)	**96.34 ± 0.16**	**96.29 ± 0.35**	**92.70 ± 0.67**	**99.30 ± 0.16**	**99.27 ± 0.16**	**98.60 ± 0.32 **

Note: The bold values indicate the best values.

**Table 7:** Prediction results of methods on PPI network datasets.

	DF-PPI (Ours)	LightGBM-PPI (2020)	GTB-PPI (2020)	GcForest-PPI (2021)	SDNN-PPI (2022)	EResCNN (2023)
CD9	**16/16**	**16/16**	15/16	**16/16**	**16/16**	**16/16**
Wnt	**96/96**	89/96	92/96	94/96	**96/96**	90/96
Cancer	**108/108**	None	None	**108/108**	**108/108**	107/108

Note: the prediction results of the other methods are published in the authors’ articles with the classification threshold chosen as 0.5. The bold values indicate the best values.

## Data Availability

The datasets and source codes are available at https://gitlab.com/nhanth/DF-PPI.git (accessed on 27 March 2024).

## Abbreviations

The following abbreviations are used in this manuscript:

PPI	Protein–Protein Interaction
APAAC	Amphiphilic Pseudo-Amino Acid Composition
LD	Local Descriptor
C, T, D	Composition, Transition, Distribution
PseAAC	Pseudo-Amino Acid Composition
PSSM	Position-Specific Score Matrix
ML	Machine Learning
DL	Deep Learning
CNN	Convolutional Neural Network
RNN	Recurrent Neural Network
GRU	Gated Recurrent Unit
MLP	Multilayer Perceptron
NLP	Natural Language Processing
SGD	Stochastic Gradient Descent

## Appendix A

**Table A1:** Seven groups follow the dipoles and volume of the side chains of 20 amino acids.

Group Index	Amino Acid
Group 1	Alanine (A), Glycine (G), Valine (V)
Group 2	Cysteine (C)
Group 3	Aspartic acid (D), Glutamic (E)
Group 4	Phenylalanine (F), Proline (P), Isoleucine (I), Leucine (L)
Group 5	Histidine (H), Glutamine (Q), Asparagine (N), Tryptophan (W)
Group 6	Lysine (K), Arginine (R)
Group 7	Methionine (M), Serine (S), Tyrosine (Y), Threonine (T)

**Table A2:** Top five patterns of permuting amino acids and grouping them into four groups **$G_{0}$**, **$G_{1}$**, **$G_{2}$** and **$G_{3}$**.

Group Index	$G_{0}$	$G_{1}$	$G_{2}$	$G_{3}$
$g_{1}$	$\left\{p_{1},p_{2},p_{3},p_{4}\right\}$	$p_{5}$	$p_{6}$	$p_{7}$
$g_{2}$	$\left\{p_{1},p_{2},p_{3},p_{5}\right\}$	$p_{4}$	$p_{6}$	$p_{7}$
$g_{3}$	$\left\{p_{1},p_{2},p_{3},p_{6}\right\}$	$p_{4}$	$p_{5}$	$p_{7}$
$g_{4}$	$\left\{p_{1},p_{2},p_{3},p_{7}\right\}$	$p_{4}$	$p_{5}$	$p_{6}$
$g_{5}$	$\left\{p_{1},p_{2},p_{4},p_{5}\right\}$	$p_{3}$	$p_{6}$	$p_{7}$
